# Dauer larva-derived extracellular vesicles extend the life of *Caenorhabditis elegans*

**DOI:** 10.1007/s10522-023-10030-5

**Published:** 2023-04-13

**Authors:** Jing Ma, Yi-ting Wang, Ling-hui Chen, Bang-ya Yang, Yong-zhu Jiang, Lan-xi Wang, Zhi-qi Chen, Guan-rong Ma, Liao-qiong Fang, Zhi-biao Wang

**Affiliations:** 1 State Key Laboratory of Ultrasound in Medicine and Engineering, College of Biomedical Engineering, Chongqing Medical University, Chongqing, 400016 China; 2 National Engineering Research Center of Ultrasound Medicine, Chongqing, 401121 China

**Keywords:** *C. elegans*, Dauer, Extracellular vesicles (EVs), Lifespan

## Abstract

There is growing evidence that extracellular vesicles (EVs) play a functional role in tissue repair and anti-aging by transferring the contents of donor cells to recipient cells. We hypothesized that Dauer (*C. elegans*), known as “ageless” nematodes, can also secrete extracellular vesicles and influence the lifespan of *C. elegans*. Here, we isolated EVs of dauer larvae (dauer EVs). Dauer EVs were characterized using transmission electron microscopy, nanoparticle tracking analysis (NTA), and Western blot analysis. Wild-type *C. elegans* were fed in the presence or absence of dauer EVs and tested for a range of phenotypes, including longevity, mobility and reproductive capacity. Results showed that dauer EVs increased the average lifespan of nematodes by 15.74%, improved mobility, slowed age-related pigmentation as well as body length, and reduced the accumulation of reactive oxygen species and lipids, while not impairing nematode reproductive capacity. These findings suggest that dauer EVs can extend the lifespan of *C. elegans* as well as the healthy lifespan by reducing ROS accumulation, with potential anti-aging capacity.

**Supplementary Information:**

The online version contains supplementary material available at 10.1007/s10522-023-10030-5.

## Introduction

Aging is a process involving the degeneration of physiological functions. Over time, this process eventually leads to death of the body as various diseases emerge. Aging is an irreversible process, but with the study of aging mechanisms, it has been revealed that the rate of aging can be regulated by various genetic and environmental manipulations (Zeng et al. 2020). Changing the rate of aging of the body is one strategy for increasing life expectancy while reducing the probability of disease. Therefore, there is a growing need for strategies to slow the aging process and prevent aging-related diseases (Shetty et al. 2018).

Extracellular vesicles (EVs) have been extensively studied as messengers of intercellular communication. EVs from stem cells, young individuals, and long-lived individuals have been found to play important roles in tissue repair and anti-aging strategies (Fafián-Labora et al. 2020; Roefs et al. 2020). Stem cell-derived EVs inherit the ability of stem cells to rescue target cells. Researchers revealed that triple-helix repeat-containing 1 and osteoprotegerin from urine-derived stem cell-derived EVs reduced age-related bone loss (Chen et al. 2019). EVs from human umbilical cord mesenchymal stem cells, adipose stem cells, and induced multifunctional stem cells carry antioxidant proteins (glutathione peroxidase 1, superoxide dismutase, and catalase) that influence cell proliferation, collagen biosynthesis, DNA damage repair by controlling mitochondrial membranes and scavenging reactive oxygen species (ROS) accumulated during aging, thereby exerting anti-photoaging effects (Choi et al. 2019; Deng et al. 2020; Oh et al. 2018). Further studies revealed that EVs isolated from the blood of young donors contained high levels of antioxidant proteins (nicotinamide phosphoribosyltransferase and glutathione-related protein) that facilitate the anti-aging treatment of target tissues (hypothalamus, pancreas, retina, and hippocampus) by enhancing NAD + biogenesis. The proteins also enhanced endogenous glutathione S-transferase activity, scavenged ROS, and prevented lipid peroxidation (Yoshida et al. 2019). EVs from long-lived individuals have higher antioxidant capacity, and they can alleviate chronic inflammation caused by aging (Storci et al. 2019). These results suggest that source-specific EVs can achieve the goals of repairing tissue damage, delaying aging, and extending longevity.

Almost all cells, especially eukaryotic cells, secrete EVs to the outside environment. The EVs of nematodes were isolated and purified using sterile liquid culture for 24 h. Nematode EVs were found to be associated with immunity and senescence in a histological study (Russell et al. 2020a, b). The nematode life cycle consists of the embryonic stage, four larval stages (L1–L4), and the adult stage. Under unfavorable conditions, larvae enter the dauer stage at L2, which allows them to survive for several months without feeding, and when conditions become favorable again, they end the dauer state and re-enter their life cycle from the L4 stage without affecting their subsequent life course (Karp & Greenwald 2013; Wirick et al. 2021). Dauer larvae are also known as “ageless” nematodes, and dauer nematodes are in the juvenile stage of the nematode life cycle (Ewald et al. 2018).

Over past decades, *Caenorhabditis elegans* has become one of the most typical animal models in the field of aging. As a multicellular organism, its short life cycle, transparent genetic characteristics, and various physiological and behavioral phenotypes that change with age make *C. elegans* an excellent in vivo model for studying the environmental and genetic basis of lifespan. Some of the seminal findings in the study of aging were first discovered in nematodes and later validated in other advanced species; for example, insulin/IGF‐1 signaling (IIS), which also demonstrated that signaling pathways associated with aging are highly conserved in evolutionary terms (Kenyon et al. 1993). In summary, *C. elegans* plays an important role as a “transit point” for the study of the basic biology of aging and the translational application of anti-aging strategies. Most current studies on the use of EVs for delaying aging and extending lifespan have been conducted at the tissue level because it is difficult to simply visualize the effects of EVs on the organism because of the long lifespan of mammals, and the complexity of the in vivo environment. Therefore, we propose to use L4 stage *C. elegans* and the EVs of dauer larvae as experimental subjects to investigate the effects of EVs on nematode senescence and longevity.

Based on prior findings, we hypothesized that dauer larvae secrete EVs and that these EVs have anti-aging-related effects. In this study, the role of dauer EVs in nematode longevity was investigated in L4 *C. elegans*. Dauer EVs induced dose-dependent increases in longevity. Healthy senescence-related phenotypes and motility were improved at optimal doses, whereas reproductive capacity was not significantly changed. Further testing of aging-related indicators revealed age-dependent improvements in lipofuscin accumulation, ROS levels, and adiposity levels at the optimal dose, tentatively suggesting that dauer EVs-mediated lifespan extension in nematodes is related to antioxidant capacity. The results of this study demonstrate the great potential of dauer EVs for extending the healthy lifespan, which will contribute to the further development of EVs for promoting healthy lifespan extension in individuals and provide ideas for studying the anti-aging effects of EVs.

## Materials and methods

### Strain, culture and handing procedure

The wild-type *C. elegans* strain N2 (ancestral) and uracil auxotroph *Escherichia coli* strain OP50 were obtained from the Caenorhabditis Genetics Center (University of Minnesota, Minneapolis, MN, USA). Nematode growth medium (NGM) was supplied by Shanghai Haohai Biological Technology Co. Ltd. In addition to the acquisition of dauer larvae, the other worms were cultured and analyzed on solid medium (NGM). *E. coli* OP50 was plated in NGM dishes. All other chemicals used were of analytical reagent grade.

### Dauer larvae maintenance conditions and EV acquisition

To generate a sufficient biomass to extract EVs, we cultured a large number of dauer larvae in a liquid environment (Supplementary Methods, Dauer larvae culture) (Hibshman et al. 2021; Karp 2018; Russell et al. 2020a, b). Dauer larvae and culture supernatant were collected, dauer larvae were ground, and the supernatant was collected at 13,000 × *g* for 10 min. The supernatant of the two parts was filtered at 0.22 μm, and the precipitate was collected at 100,000 × *g* for 90 min (Beckman, US) (Crescitelli, et al. 2021). The collected precipitates were analyzed by electron microscopy, and the size of the precipitate was determined by nanoparticle tracking (Nano Sight 300, Malvern Panalytical, UK). Finally, the EVs marker CD-63 (anti-CD63, Abcam, UK) and membrane vesicle protein marker LMP-1 (anti-LMP-1, Developmental Studies Hybridoma Bank, USA) were detected by western blotting (Huynh et al. 2016; Kostich et al. 2000) to confirm that the final collected precipitate contained dauer EVs.

### Lifespan assay

All lifespan tests were performed at 20 °C. NGM Petri dishes were prepared according to the instructions provided by the reagent company. NGM medium was sterilized at 120 °C, and additives containing cholesterol and streptomycin were added after the medium was cooled to 55 °C. NGM dishes were coated with 30 µl of *E. coli* OP50 and incubated for 8 h at room temperature before starting the assays. Synchronized L4 larvae (3 days following hatching) were transferred together with various concentrations of dauer EVs to 35-mm Petri dishes at 20 °C and then transferred to NGM dishes 2 h later (Fueser et al. 2021). The day of nematodes treatment was defined as day 0. These nematodes were observed daily and were considered dead if they did not respond to physical stimuli. Nematodes that were missing or hatching inside the eggs were not included in the statistics (Maures et al. 2014). The experiment was repeated three times independently with at least 30 worms in each group.

### Motility

Body bending is defined as a shift of one wavelength relative to the long axis of the body. To measure the rate of body bending, the number of body bends in nematodes was recorded over 10 s after transferring 20 µl of S-basal droplets on days 2, 5, and 8 with and without dauer EVs treatment (Nourse et al. 2021). The experiment was repeated three times independently with 10 nematodes in each group.

### Body length change experiment

To measure changes in nematode body length, nematodes treated with or without 1 × 10^7^ particles/ml dauer EVs were photographed on 2% agar after anesthesia on days 5, 8, and 11, and their body length was quantified using ImageJ (Hong et al. 2022). The experiment was repeated three times independently with at least 20 nematodes in each group.

### Lipofuscin accumulation assay

To study lipofuscin accumulation in nematodes treated with 1 × 10^7^ particles/ml dauer EVs, treated or untreated nematodes were cultured for 5, 10, or 15 days; anesthetized with levamisole ((S)-2,3,5,6-tetrahydro-6-phenylimidazo(2,1-b) thiazole, Aladdin, China); and observed under a fluorescence microscope (Leica Microsystems, Germany) (Lin et al. 2019). The lipofuscin accumulation experiment was repeated three times. The images were analyzed using ImageJ software, and the total number of nematodes was counted. At least 20 nematodes were used in each group.

### Reproduction assay

To determine the effect of 1 × 10^7^ particles/ml dauer EVs on nematode oviposition, at least 10 nematodes were cultured and treated as described in the lifespan experiment. Ten nematodes (in triplicate) were randomly selected and transferred to fresh NGM dishes on the third day of culture (oviposition period) and allowed to lay eggs for 72 h (Yang et al. 2018).

Similarly, to determine the effect of dauer EVs (1 × 10^7^ particles/ml) on age-associated vulval integrity defects (AVID), treatments were performed as described in the lifespan experiment. The AVID phenotype was observed and determined during aging until all nematodes died. The experiment was independently repeated three times with at least 20 nematodes in each group.

### Effects of Dauer EVs on ROS accumulation of *C. elegans*

ROS levels were measured using 2′,7′-dichlorofluorescein diacetate (H2DCF-DA, Meilunbio, China). The nematodes were collected on day 8 after 1 × 10^7^ particles/ml dauer EVs treatment with the same pre-treatment utilized in the lifespan experiment (Liu et al. 2022). The nematodes were collected, treated with H2DCF-DA, and observed under a fluorescent microscope. The experiment was repeated three times independently. Images were analyzed using ImageJ software with at least 20 nematodes per group.

### Fat accumulation assay

To study lipid accumulation after 1 × 10^7^ particles/ml dauer EVs treatment, nematodes were fixed in 1% paraformaldehyde after washing on the second and eighth days after EVs treatment and then stained at room temperature using a mixture of 2% oil red O (Bio-sharp, China) and 2% Triton X-100 in equal proportions, and the staining was observed under a fluorescence microscope and photographed (Shen et al. 2019). The experiment was repeated three times independently. The pictures were analyzed using ImageJ software with at least 20 nematodes in each group.

### Statistical analysis

We used GraphPad Prism 8.0 to conduct statistical analyses. For lifespan experiments, the Kaplan–Meier method was utilized, and P values were calculated using the log-rank test. To compare two groups, Student’s *t*-test was performed. *P* < 0.05 was considered statistically significant.

## Results

### Dauer larvae secrete EVs carrying CD-63 and LMP-1 proteins

**Fig. 1:**
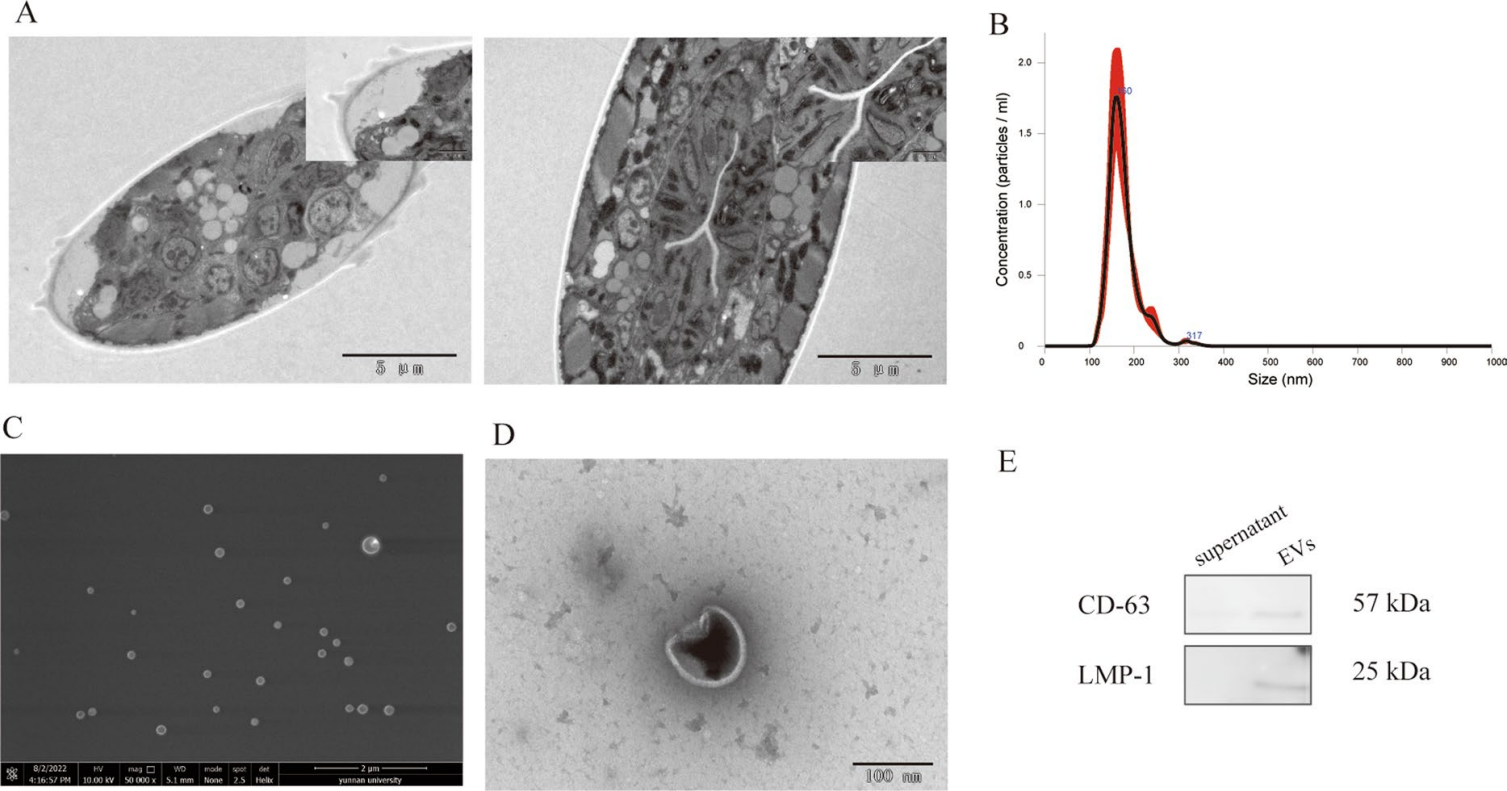
Characterization of the EVs of dauer larvae. **A** Representative cross-sectional transmission electron microscopy images of dauer larvae, in which a distinctly thickened cuticle with a closed oral structure was visible. Scale bar = 5 µm; local magnification, × 5. **B** Nanoparticle tracking analysis of dauer EVs with a peak particle size of approximately 160 nm. **C** Scanning electron microscopy of dauer EVs. The nanoparticles were visible under the microscope, exhibiting a distinct spherical structure and a size range of 100–200 nm. Scale bar = 2 µm. **D** Transmission electron microscopy analysis of dauer EVs. The particles were subjected to uranyl acetate staining, and the lipid bilayer structure was clearly visible under the microscope. Scale bar = 100 nm. **E** Western blotting of dauer EVs and supernatant from wash. Anti-CD-63 and anti-LMP-1 antibodies showed strong immunogenicity at the expected sizes in the EVs group, while no strong immunogenicity was observed in the control group, the wash supernatant group

We examined cross-sections of nematodes using transmission electron microscopy (TEM) and found closed mouths, thickened cuticles, and dauer alae (Fig. 1A). To determine the number of obtained particles and their size distribution, nanoparticle tracking analysis (NTA) was performed. To maintain the concentration of particles within the operating range of Nano Sight 300, we diluted the samples at a ratio of 1:100 using S-basal before analysis and detection. Three replicates were examined, and they were found to have a dominant monodisperse particle population with an average size of 160 nm (Fig. 1B). Then, the separated particles were detected by scanning electron microscopy (SEM) and TEM. An obvious spherical structure was observed by SEM, and an obvious bilayer structure was observed by TEM (Fig. 1C and D). CD63 is a membrane protein recognized as a marker of eukaryotic outer membrane vesicles, and LMP-1 is a nematode homolog of the common mammalian outer membrane vesicle membrane protein LAMP-1 (Kowal et al. 2016; Vonk et al. 2018). Therefore, we performed western blotting of the isolated pellets using monoclonal antibodies against CD63 and LMP-1 and found strong immunoreactivity in the isolated pellets (Fig. 1E). Combined with the electron microscopy, NTA, and western blotting data, our isolated pellet was confirmed to contain dauer EVs.

### EVs extend the lifespan of *C. elegans*

**Fig. 2:**
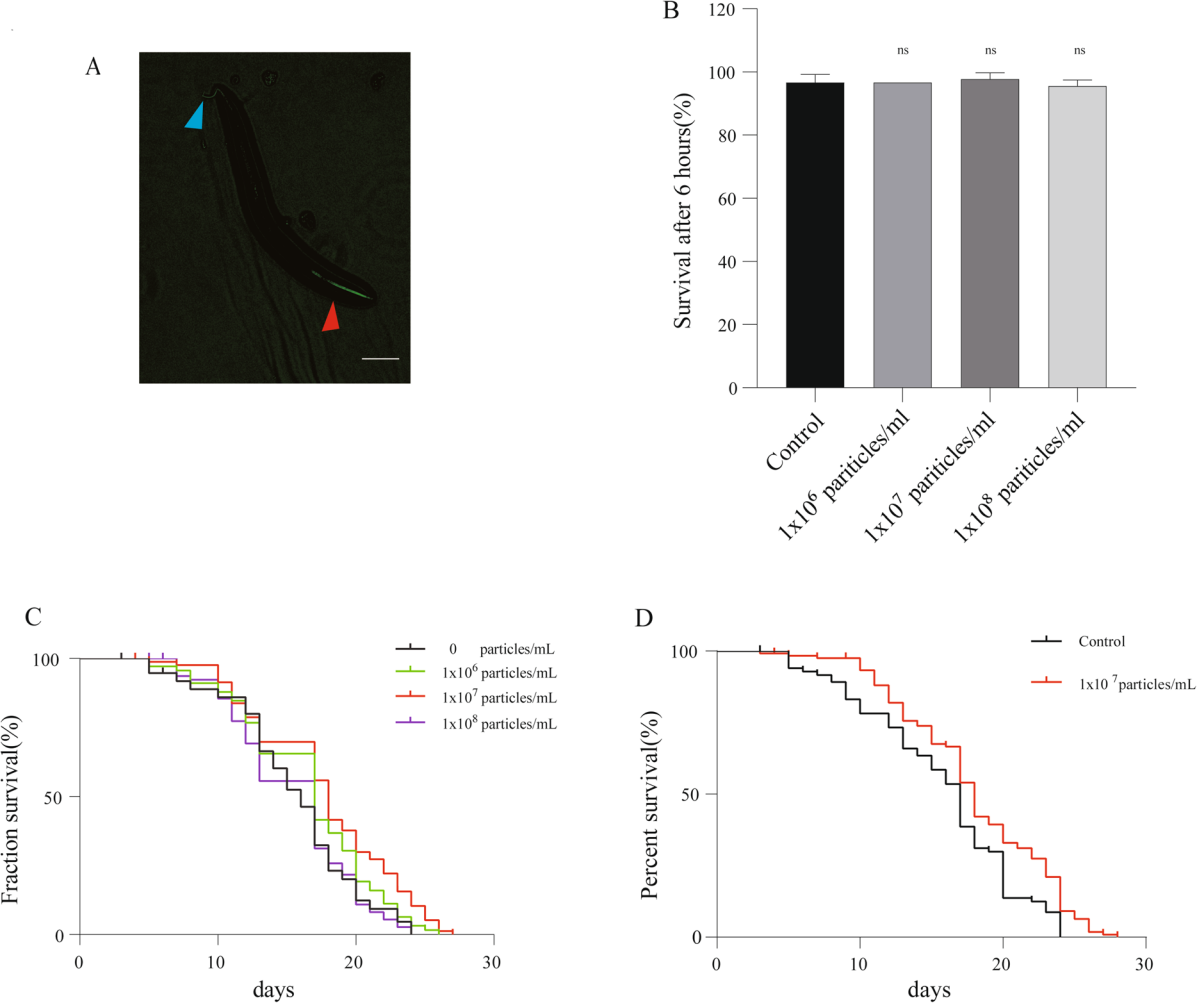
Toxicity assessment and longevity analysis of different doses of dauer EVs in wild-type N2 worms. **A** Intake study of dauer EVs. DIO-labeled dauer EVs were introduced into the nematode intestine through the oral cavity via the pharyngeal activity of the nematodes. The red arrow is the head of the nematode and the blue arrow is the tail of the nematode. **B** Toxicity analysis of dauer EVs. The survival rates of nematodes following exposure to dauer EVs (1 × 10^6^, 1 × 10^7^, and 1 × 10^8^ particles/ml) for 6 h were not significantly different from the control, illustrating the absence of a significant toxic effect. **C** Lifespan analysis of nematodes in the presence of dauer EVs (1 × 10^6^, 1 × 10^7^, and 1 × 10^8^ particles/ml). **D** Lifespan test of nematodes at the optimal dose (1 × 10^7^ particles/ml). For panel (**A**), comparisons were performed using one-way ANOVA followed by the post-hoc Bonferroni test. Lifespan analysis was performed using the Kaplan–Meier method. Data are displayed as the mean ± SEM. Data were analyzed using an independent-samples *t*-test (ns = non-significant)

To fully investigate the effect of dauer EVs on the longevity of *C. elegans*, we first explored whether dauer EVs could enter nematodes using the fluorescent membrane dye DIO (Supplementary Methods, Dauer EVs intake experiment), and green fluorescence-labeled dauer EVs were detected in the nematode oral cavity and anterior part of the intestine 2 h after exposure, indicating that dauer EVs entered the nematode oral cavity upon feeding and the nematode intestine upon pharyngeal pumping activity (Fig. 2A). We then treated type N2 *C. elegans* at the L4 period using three different doses of vesicles (1 × 10^6^, 1 × 10^7^, and 1 × 10^8^ particles/ml; Supplementary Methods, Dauer EVs toxicity test). The results illustrated that these EV doses were not lethal to the nematodes within 6 h, indicating that short-term treatment with dauer EVs was not significantly toxic to N2 *C. elegans* (Fig. 2B).

Immediately afterwards, we examined the effect of the three EVs doses on the lifespan of nematodes after 2 h. Treatment with dauer EVs (1 × 10^7^ particles/ml) increased the mean lifespan of nematodes by 15.74% versus the control (17.747 ± 5.259 days vs. 15.333 ± 4.808 days, *P* = 0.001). Conversely, dauer EVs treatment at doses of 1 × 10^6^ and 1 × 10^8^ particles/ml had no significant effect on lifespan versus the control (Table S1 and Fig. 2C).

### EVs improve the healthy lifespan of *C. elegans*

**Fig. 3:**
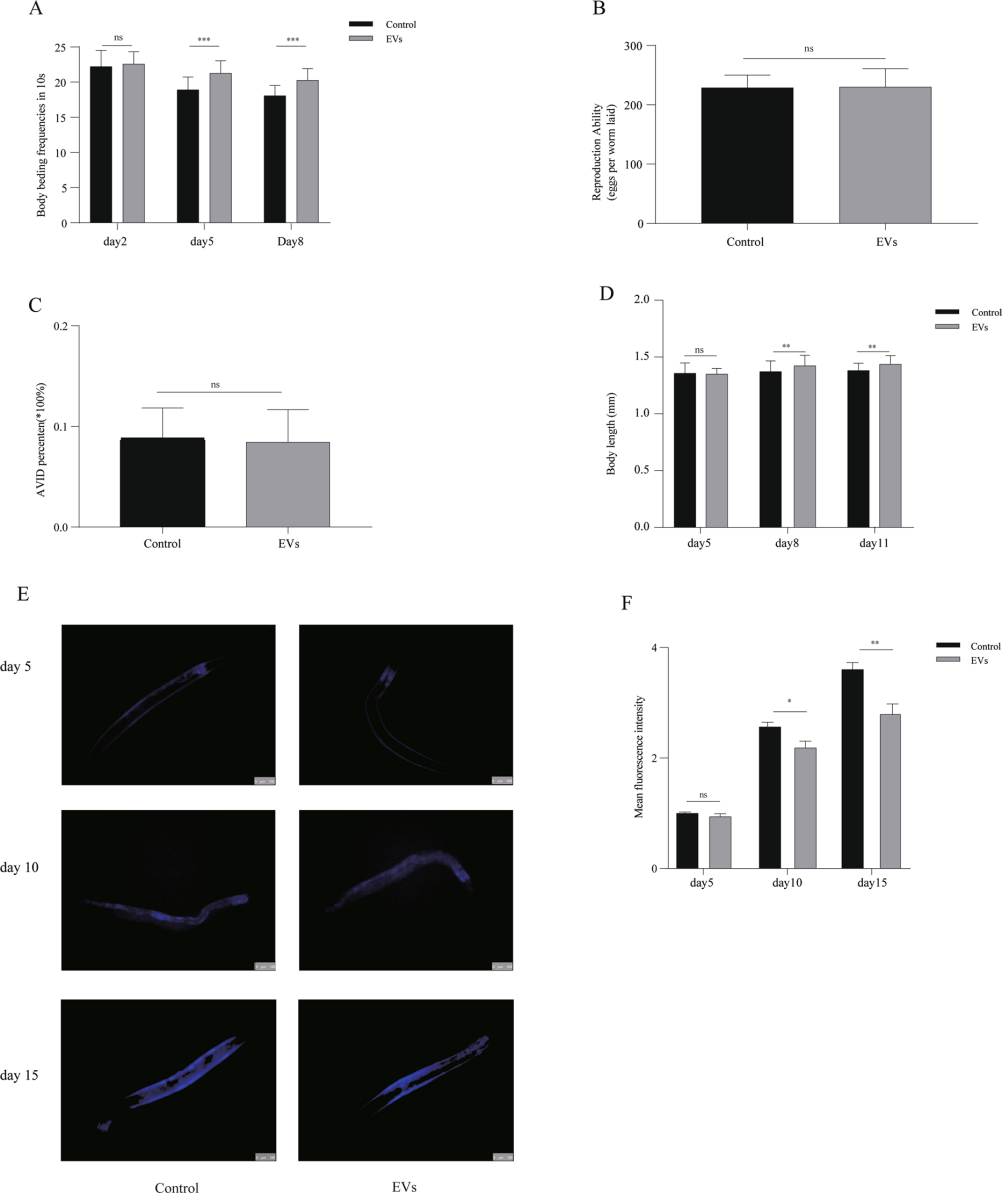
Effects of dauer EVs (1 × 10^7^ particles/ml) on nematode physiological functions and biomarkers of aging (lipofuscin). **A** Effect of dauer EVs on nematode motility. The locomotor ability of nematodes, which gradually decreased with age, was better in the EVs group than in the control group. **B** Egg production was not significant difference within 72 h after dauer EVs treatment compared to that in the control group, and the AVID phenotype did not improve throughout the life span following dauer EVs treatment. **C** Dauer EVs increased the lifespan of nematodes without reducing reproductive capacity. **D** Body length did not differ between the EVs and control groups in the early stage (day 5), but in the middle and late stages (days 8 and 11, respectively), dauer EVs-treated nematodes had a significantly longer body length than control nematodes. **E** Representative fluorescence images of lipofuscin. Scale bar = 100 µm. **F** Intestinal lipofuscin levels were significantly lower in the middle and late stages (days 10 and 15, respectively) after dauer EVs treatment (1 × 10^7^ particles/ml) compared to the control findings. Lipofuscin levels were quantified using ImageJ software. All analyses were performed using an independent-samples *t*-test. (*P < 0.05, **P < 0.005, ***P < 0.0005, ns = non-significant)

The aging process affects the longevity and healthy lifespan of nematodes, and healthy lifespan can be assessed by functional declines in performance tests (Bansal et al. 2015). We studied behavioral and functional phenotypes closely related to aging, including motility and reproductive capacity (Son et al. 2019). The mean lifespan of nematodes at the optimal EVs dose was increased by 14.441% versus the control (17.901 ± 9.567 days vs. 15.642 ± 8.6692 days, *P* = 0.002, Fig. 2D), in general agreement with the previous results. The first phenotype that we tested was the nematode bending rate, which reflects the nematode’s motility and gradually decreases with the aging process (Li et al. 2021). We measured the number of nematode bends per 10 s on days 2, 5, and 8. The numbers of body bends per 10 s on days 2, 5, and 8 in control nematodes were 22.2 ± 2.280 freq, 18.9 ± 1.807 freq, and 18.068 ± 1.437 freq, respectively, versus 22.567 ± 1.736 freq, 21.267 ± 1.741 freq, and 20.267 ± 1.660 freq, respectively, after dauer EVs treatment (all *P* < 0.0001). The results demonstrated that dauer EVs improved this phenotype (Table S2 and Fig. 3A). The second tested phenotype was the nematode reproductive capacity, which is believed to be closely related to longevity. The assessment of nematode oviposition and AVID phenotypes is an important basis for determining whether dauer EVs impair nematode reproductive capacity (Leiser et al. 2016; Lenz, et al. 2019). As presented in Fig. 3B and C, there was no significant difference in egg production between dauer EVs-treated and untreated nematodes at 72 h (*P* = 0.228) and no prevention of the senescence-induced AVID phenotype (*P* = 0.177), indicating that dauer EVs-induced longevity is not dependent on reproductive signaling pathways.

A longer lifespan is often associated with nematode growth capacity (Liao et al. 2011; Hong et al. 2022). To understand whether dauer EVs affect nematode growth, we measured the change in nematode body length as an indicator of the adult lifespan (Hong et al. 2022). On day 5 after treating the nematodes with EVs, body length was not significantly different between the control and EVs groups (*P* = 0.681). However, treated nematodes were significantly longer than untreated nematodes on days 8 (1.404 ± 0.081 mm vs. 1.389 ± 0.071 mm, *P* = 0.009) and 11 (1.438 ± 0.074 mm vs. 1.382 ± 0.063 mm, *P* = 0.003, Fig. 3D).

Lipofuscin is an autofluorescent substance that accumulates gradually with the age of *C. elegans* (Yu et al. 2021). We measured the in vivo autofluorescence intensity of lipofuscin on days 5, 10, and 15 of nematode growth, and the results indicated that lipofuscin accumulated gradually over time. However, lipofuscin accumulation in dauer EVs-treated nematodes was decreased relative to the control by 6.167% on day 5 (*P* = 0.117), 14.782% on day 10 (*P* = 0.010), and 22.620% on day 15 (*P* = 0.003, Fig. 3E and F).

### EVs reduce in vivo ROS levels in *C. elegans*

**Fig. 4:**
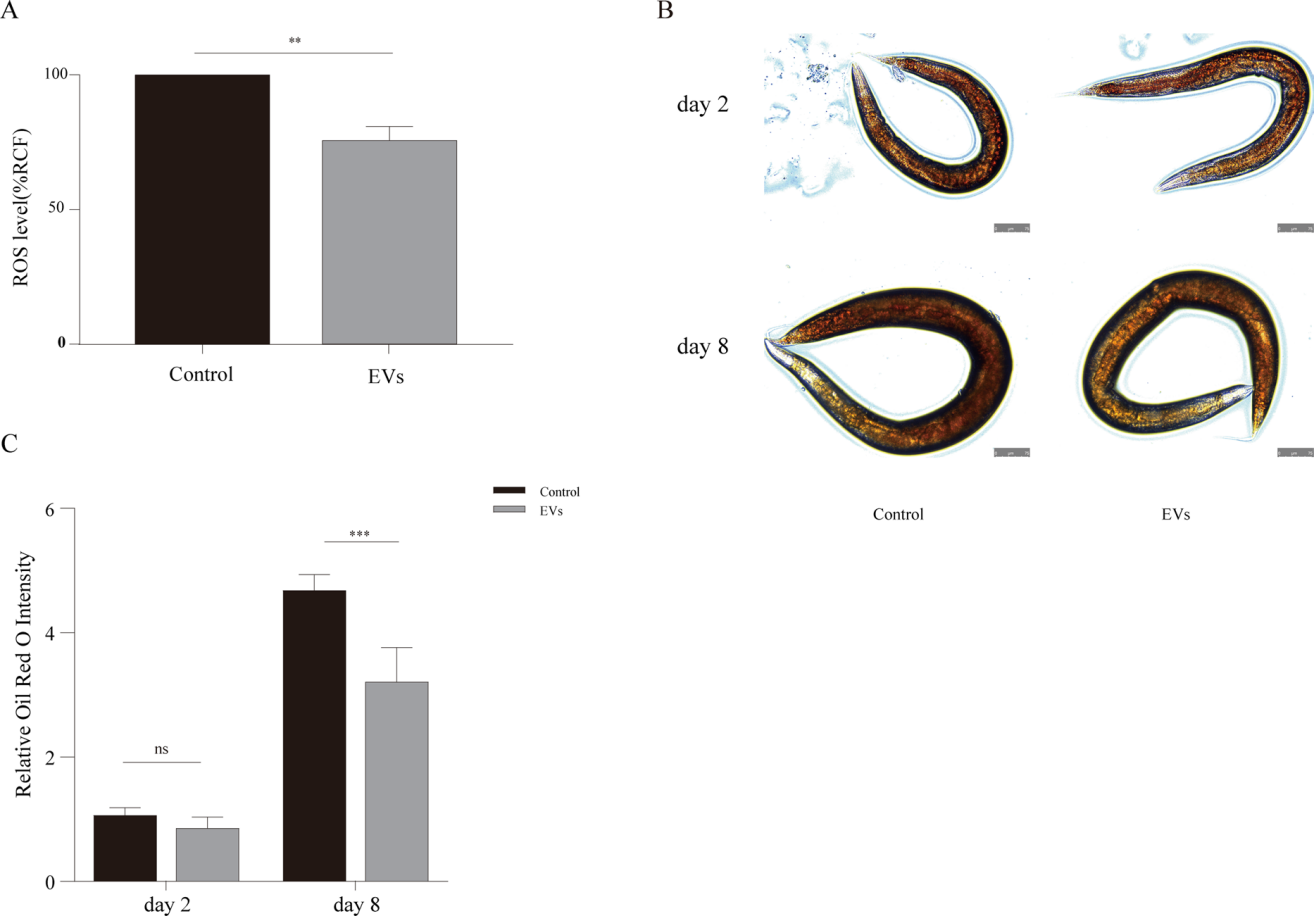
Effect of dauer EVs (1 × 10^7^ particles/ml) on reactive oxygen species (ROS) and fat accumulation in nematodes. **A** ROS levels in nematodes were measured using 2′,7′-dichlorofluorescein diacetate staining on day 8, and significantly reduced ROS accumulation was observed in the EVs group compared to the control level. **B** Representative microscopic images of oil red O staining. Scale bar = 100 µm. **C** A significant decrease in nematode fat levels on day 8 was observed in the EVs group compared to the findings in the control group. ROS and fat levels were quantified using ImageJ software. Data are presented as the mean ± SEM. Data were analyzed using an independent-samples *t*-test (*P < 0.05, **P < 0.005, ***P < 0.0005)

To investigate whether the mechanism of lifespan extension in dauer EVs is related to ROS levels in *C. elegans*, we compared ROS levels in treated and untreated *C. elegans* using the ROS probe H2DCF-DA. ROS accumulation was reduced in worms treated with 1 × 10^7^ particles/ml dauer EVs (*P* = 0.001, Fig. 4A). This suggests that the increased lifespan of dauer EVs-treated *C. elegans* is partly attributable to decreased ROS accumulation.

### EVs reduce lipid accumulation in nematodes

In wild-type *C. elegans*, increased ROS production might lead to excessive lipid accumulation (Hughes et al. 2007; Wang et al. 2018, 2022). The results revealed no significant difference in body fat levels in nematodes on day 2 (*P* = 0.172), whereas on day 8, dauer EVs-treated nematodes exhibited significantly lower body fat levels than control nematodes (*P* = 0.0004, Fig. 4B and C).

## Discussion

The aging process is accompanied by a decline in the physiological functions of the organism, and it is the most important factor for the development of many diseases in later life (Benito-Cuesta et al. 2021). A number of structures involved in intercellular communication (including EVs) have been identified as effective anti-aging factors in studies of anti-aging programs (Yin et al. 2021).

This is the first study on the effects dauer EVs on longevity in *C. elegans*. Therefore, we first identified the obtained larvae to ensure that the extracted EVs were derived from dauer larvae, and based on their unique physiology (closed oral cavity, thickened cuticle), electron microscopic observation revealed that the obtained nematodes were dauer larvae. We extracted EVs from nematode tissues and liquid medium and identified membrane vesicles at three levels: size, morphology, and molecular characterization.

When EVs enter the organism and act on target cells, it is first necessary to determine whether they are biotoxic. We first investigated nematode mortality following exposure to EVs at doses of 1 × 10^6^, 1 × 10^7^, and 1 × 10^8^ particles/ml. The results demonstrated that no significant biological toxicity occurred when nematodes were exposed to dauer EVs for a short period (6 h). Immediately afterward, we performed lifespan analysis at the aforementioned doses. Dauer EVs at 1 × 10^7^ particles/ml most effectively prolonged the nematode lifespan, whereas the other doses had no significant effect. Previous reports of lifespan extension by plant molecules suggest a dose dependence of nematode lifespan extension, with nematode lifespan showing a bell shape at different doses our results are generally consistent (Powolny et al. 2011). Life extension is not significant at lower doses (1 × 10^6^ particles/ml), possibly when the concentration is lower than the effective dose. On the other hand, under high concentration conditions(1 × 10^8^ particles/ml), a non-significant shortening of nematode lifespan was observed, and high concentrations of dauer EVs may have reduced the involvement of key factors involved in lifespan extension thus inducing feedback regulation (Srivastava et al. 2017; Pandey et al. 2019). However, these findings need to be further explored.

The advantages of the *C. elegans* model in the field of aging as well as anti-aging research have been well documented (Shen et al. 2018). As biological individuals, aging-related indicators respond to the lifespan as well as the rate of aging of the organism and the length of the healthy lifespan. Aging is accompanied by changes in various physiological indicators. Therefore, *C. elegans* provides a good opportunity to identify biological and pharmacological interventions for anti-aging. Based on the *C. elegans* model, we systematically evaluated the effect of dauer EVs on the healthy lifespan. We found that these EVs prolonged the nematode lifespan without affecting nematode reproduction and grow. Instead, dauer EVs significantly enhanced nematode motility. We also found that age-related pigmentation changes were effectively alleviated by dauer EVs treatment, especially in the middle and late stages. These findings suggest that dauer EVs have the potential to improve the healthy lifespan, a hypothesis that was further confirmed by an in-depth study of ROS accumulation.

ROS are highly reactive oxygen molecules that accumulate with age, and they can cause genotoxicity and physiological damage, which are important factors that contribute to aging (Back et al. 2010). Excessive ROS levels can lead to DNA damage, alter gene expression, disrupt cell signaling, affect normal mitochondrial function, and ultimately lead to the disruption of normal cellular physiological functions and death (Labuschagne & Brenkman 2013; Zhang et al. 2022). We measured ROS levels in both groups of nematodes on day 8 and observed a decrease in ROS levels in nematodes after dauer EVs treatment, suggesting that dauer EVs are extend the nematode lifespan by reducing ROS accumulation.

According to existing studies, the redistribution and abnormal accumulation of fat in *C. elegans* during the aging process might lead to the toxic effects of adiposity and a decline in tissue function associated with aging (Savini et al. 2013). At the same time, excessive ROS accumulation can also lead to excessive fat accumulation, which is one of the causes of obesity (Wang et al. 2018). Therefore, after observing that dauer EVs mediated the reduction of ROS levels, we measured adiposity levels in nematodes. We measured the fat level in nematodes on days 2 and 8, and the nematode fat level was significantly lower in the dauer EVs group than in the control group on day 8, whereas no difference was noted between the groups in the early stage.

Our results suggest that dauer EVs prolong the nematode lifespan by reducing ROS levels without compromising normal nematode physiological functions, especially nematode reproductive capacity, but to some extent, dauer EVs alleviated the reduction of motility, accumulation of lipofuscin, and changes of body length associated with aging, thereby increasing the healthy nematode lifespan. Further studies will aim to trace the pathways by which dauer EVs affect ROS levels and determine whether their anti-aging mechanism can be replicated in other mammalian models. Our work provides an idea for studying EVs for expanding the individual lifespan as well as in vivo models of healthy lifespan extension.

## Supplementary Information

Below is the link to the electronic supplementary material.

Supplementary file1 (DOCX 22 KB)

## Data Availability

The data that support the findings of this study are available from the corresponding author upon reasonable request.
