# Supplementary material for: Dauer larva-derived extracellular vesicles extend the life of Caenorhabditis elegans
Source: Biogerontology. 2023 Apr 13;24(4):581–92. doi: 10.1007/s10522-023-10030-5 (PMC10267011; doi:10.1007/s10522-023-10030-5)
Supplement: Supplementary file 1 — Supplementary file1 (DOCX 22 KB) [file 10522_2023_10030_MOESM1_ESM.docx]

Supplementary information

**The Dauer Larva derived Extracellular Vesicle extend the life of Caenorhabditis elegans via reducing ROS accumulation**

Jing Ma^a^, Yiting Wang^a^, Linghui Chen^a^, Bangya Yang^a^, Yongzhu Jiang^a^, Lanxi Wang^a^, Guanrong Ma^a^, Zhiqi Chen^a^, Liaoqiong Fang^a,b, *^, Zhibiao Wang^a,b, *^.

^a^ State Key Laboratory of Ultrasound in Medicine and Engineering, College of Biomedical Engineering, Chongqing Medical University, Chongqing, 400016, China.

^b^ National Engineering Research Center of Ultrasound Medicine, Chongqing, 401121, China.

^*^ Corresponding author. Liao-qiong Fang, State Key Laboratory of Ultrasound in Medicine and Engineering, College of Biomedical Engineering, Chongqing Medical University, Chongqing, 400016, China. National Engineering Research Center of Ultrasound Medicine, Chongqing, 401121, China. E-mail: lqfang06@163.com

Zhi-biao Wang, State Key Laboratory of Ultrasound in Medicine and Engineering, College of Biomedical Engineering, Chongqing Medical University, Chongqing, 400016, China. E-mail: [Wangzb@cqmu.edu.cn](mailto:Wangzb@cqmu.edu.cn)

**Supplementary methods**

Dauer larvae culture

To be able to generate sufficient biomass to extract extracellular vesicles, we first grew a large number of synchro nematodes on NGM dishes seeded with E. coil OP50 and incubated at 20°C until adulthood. Then, we cleaned and collected the worms from the petri dishes. After 3-5 washing, lysate (5M NaOH, 5% sodium hypochlorite) was added to obtain a large number of eggs. The obtained eggs were quantified under a type microscope, and the eggs were resuspended with S medium at a concentration of 5/μL. The nematodes were cultured at 20℃ and 130r/min and aseptic conditions for 12 hours until L1 larvae. These L1 larvae were collected by centrifugation at 1,000 g for 5min, unhatched eggs are collected in the sediment and removed. L1 larvae were microscopically quantified and then cultured in S medium at a concentration of 5/μL, in which 1.0 mg/mL OP50 Escherichia coli was added. Dauer larvae were cultured at 20℃ and 130r/min for 4-5 days, during which no additional food was added (Hibshman et al., 2021; Karp, 2018; Russel et al. 2020).

Dauer EVs intake experiment

DIO (Beyotime, China) was prepared into a working solution of 0.5μM using DMSO and added to EVs at this concentration, and the excess unbound dye was washed off after 20 min. The DIO-labeled DAUER EVs were co-incubated with L4 nematodes, and the green fluorescence in the nematodes was observed under a fluorescence microscope (Jiang et al.,2011)

Dauer EVs toxicity test

L4 nematodes were incubated in a liquid environment with four concentrations of DAUER EVs(0,1×10^6^,1×10^7^,1×10^8^particles/mL) at 20°C for 6 h. The survival of nematodes was observed under the microscope, and nematodes that did not respond significantly to physical stimulation were considered dead. 30 nematodes were incubated in each group, and the experiment was repeated at least three times (Yang et al.,2018).

Supplementary Table S1 DAUER EVs mediated longevity in wild type *C. elegans*.

| Treatment | Mean lifespan ± S.E. | Max lifespan ± S.E. | Mean fold increase (%) | p-value |
| --- | --- | --- | --- | --- |
| 0 | 15.333±4.808 | 22.333±1.861 |  |  |
| 1×106particles/mL | 16.500±5.005 | 23.500±1.914 | 7.611% | 0.139 |
| 1×107particles/mL | 17.747±5.259 | 26.250±0.957 | 15.740% | 0.001(**) |
| 1×108particles/mL | 15.253±4.568 | 22.800±1.643 |  | 0.864 |

**p<0.005 and ***p<0.0005

Supplementary Table S2 Effect of DAUER EVS treatment on the motility of *C. elegans*.

| Time | Body bending times in 10s (Mean ± S.E.) | | Mean fold increase (%) | p-value |
| --- | --- | --- | --- | --- |
|  | Control | DAUER EVs |  |  |
| day 2 | 22.200 ± 2.280 | 22.567 ± 1.736 | 1.653 | 0.486 |
| day 5 | 18.900 ± 1.807 | 21.267 ± 1.741 | 12.524 | < 0.0005(***) |
| day 8 | 18.069 ± 1.438 | 20.267 ± 1.660 | 12.1645 | < 0.0005(***) |

**p<0.005 and ***p<0.0005

**References**

Hibshman, J. D., Webster, A. K., & Baugh, L. R. (2021). Liquid-culture protocols for synchronous starvation, growth, dauer formation, and dietary restriction of. *STAR Protocols, 2*(1), 100276

Jiang, Qi-Ying, Lai, Li-Hua, Shen, Jie ,Wang, Qing-Qing, Xu, Fu-Jian, Tang, Gu-Ping.(2011). Gene delivery to tumor cells by cationic polymeric nanovectors coupled to folic acid and the cell-penetrating peptide octaarginine, Biomaterials, 32 (2011) 7253-7262.

Karp, X. (2018). Working with dauer larvae. WormBook : the Online Review of C. Elegans Biology, 2018

Russell, J. C., Postupna, N., Golubeva, A., Keene, C. D., & Kaeberlein, M. (2020). Purification and Analysis of Caenorhabditis elegans Extracellular Vesicles. Journal of Visualized Experiments : JoVE(157)

Yang, Zhen-Zhou, Yu, Ying-Ting, Lin, Hong-Ru, Liao, De-Chun, Cui, Xiang-Huan, Wang, Hong-Bing(2018). Lonicera japonica extends lifespan and healthspan in Caenorhabditis elegans, Free Radic Biol Med, 129 (2018) 310-322.
